# The Effort to Secure a Room in the Capitol for the Use of the Texas State Medical Association

**Published:** 1888-05

**Authors:** 


THE EFFORT to secure a room in the new Capitol for the use of the Texas State Medical Association, would have succeeded, had there been a few more days of the session. Everything was in shape, and favorable, but adjournment took place before action could be had on the joint resolution which had been adopted, setting aside a room, and which resolution had been reported on favorably, with the recommendation of the committee that it "do pass." Dr. F. R. Martin, the efficient chairman of the committee appointed by the State Medical Association to memorealize the Legislature on the subject, discharged his duty faithfully and admirably. Beginning work immediately on adjournment at Galveston, he drew up the petition, got it signed by his collegues, and presented it. He remained at Austin three days, working incessantly on the members, and finally got the bill (which, as stated, had been reported back favorably,) into the hands of Dr. Camp, with the assurance that he would get it before the House, if possible. It must be remembered that this was on the eve of adjournment, and on the eve of the great dedication, a time of excitement, and it was difficult to do anything. Speaker Pendleton had promised Dr. Martin that he would "recognize" Dr. Camp the moment he arose for the purpose of introducing the bill, and would favor a suspension of the rules to facilitate its passage. Thus, all was lovely. But on the introduction of the bill, "Bell, of

Cook," the "medlesome Mollie" of the House, a man who has made an unenviable reputation, or, rather, who has acquired great *notoriety*, interfered, and proposed to amend by setting aside a room for the "Bar Association," and another for the "Cattlemen," and so on. Dr. Camp thought best, under the circumstances, to withdraw the bill, and to bring it up early next session; by which time, it is to be hoped, "Bell, of Cook" will have been properly retired by a grateful constituency.

Dr. Martin deserves much credit for the faithful manner in which he carried out the instructions of the Texas State Association, and it is no fault of his that the bill did not get through.

